# Effect of miR-4270 Suppression on Migration in Hepatocellular Carcinoma Cell Line (HepG2)

**DOI:** 10.61186/ibj.3923

**Published:** 2023-05-29

**Authors:** Hassan Akrami, Hanieh Gholami, Mohammad Reza Fattahi, Mastaneh Zeraatiannejad

**Affiliations:** Gastroenterohepatology Research Center, Shiraz University of Medical Sciences, Shiraz, Iran

**Keywords:** Hepatocellular carcinoma, Matrix metalloproteinase, Metastasis, MicroRNA

## Abstract

ABSTRACT

**Background:**

Liver transplantation and surgical resection are two major strategies for treatment of HCC patients. One approach to treating HCC is the suppression of metastasis to other tissues. Herein, we aimed to study the effect of miR-4270 inhibitor on migration of HepG2 cells as well as activity of MMP these cells in order to find a strategy to suppress metastasis in future.

**Methods:**

HepG2 cells were treated with 0, 10, 20, 30, 40, 50, 60, 70, 80, and 90 nM of miR-4270 inhibitor, and then the cell viability was measured by trypan blue staining. Afterwards, cell migration and MMP activity of HepG2 cells were assessed by wound healing assay and zymography, respectively. The *MMP *gene expression was determined by real-time RT-PCR.

**Results:**

Results showed that miR-4270 inhibitor decreased the cell viability of HepG2 cells in a concentration-dependent manner. Also, inhibition of the miR-4270 reduced invasion, MMP activity, and expression of *MMP *genes in HepG2 cells, respectively.

**Conclusion:**

Our findings suggest that miR-4270 inhibitor decreases in vitro migration, which could help find a new approach for HCC therapy patients.

## INTRODUCTION

Liver cancer is the seventh most common cancer and the second cause of cancer mortality worldwide^[^^1^^]^. HCC with a poor prognosis and low survival rate includes about 80% of liver cancer cases^[^^2^^]^. Liver transplantation and surgical resection are two major options for HCC treatment. However, patients with advanced HCC do not respond to transplantation and surgical cures. Therefore, chemotherapy may currently be the only medical cure for the advanced HCC patients^[^^3^^]^. 

 Tissue invasion and metastasis to distant organs are important events, leading to cancer mortality^[^^4^^]^. Therefore, one strategy for cancer treatment is to inhibit angiogenesis, tissue invasion, and metastasis using chemical reagents or modify the regulation of genes and regulatory noncoding RNAs, particularly microRNAs or miRNAs^[^^5^^,^^6^^]^. MicroRNAs, a class of small non-coding RNAs, perform important roles in different cell signaling pathways associated with cell physiology such as cell cycle, apoptosis, angiogenesis, and migration, which could be considered as proper targets for the treatment of cancer patients in future^[^^6^^]^. 

 Dysregulation of miRNA expression is often related to the initiation and progression of variety types of cancers^[^^7^^,^^8^^]^. For instance, a study on microarray expression profiling of miRNAs in HCC patients reported 23 differentially expressed miRNAs in HCC^[^^9^^]^. Other studies have demonstrated differential expression of miR-4270 in HCC and other carcinomas such as gastric, lung, and breast cancers^[^^10^^]^. Aminisepehr and co-workers^[^^11^^]^ have shown that plasma miR-4270 is upregulated in breast cancer patients compared to control cases. It has also been revealed that miR-4270 is involved in different signaling pathways, including Notch signaling pathway^[^^8^^]^. These studies have explained the important function of miR-4270 in the pathogenesis of cancers and stated that modification of miR-4270 expression may lead to cancer treatment in future.

In the current study, we investigated the influence of miR-4270 inhibitor on the *in vitro* migration, MMP activity and expression of *MMP2* and *MMP9 *at RNA level in HepG2 cells. 

## MATERIALS AND METHODS

**HepG2 cell culture**

HepG2 cell line, an immortalized epithelial HCC cell line, was acquired from the National Cell bank of Iran (NCBI) affiliated to the Pasteur Institute of Iran (Tehran). HepG2 cell line was cultured in DMEM/F12 (Gibco, Belgium) supplemented with 10% (v/v) heat-inactivated FBS (Gibco, Belgium), 100 U/ml of penicillin G (Sigma–Aldrich, USA), and 100 μg/ml of streptomycin sulfate (Sigma–Aldrich) and incubated in a humidified incubator containing 95% air and 5% CO_2_ at 37 °C. 

**MiRNA inhibitor transfection**

First, 5 × 10^5^ HepG2 cells were cultured on a 60-mm^2^ plate in DMEM/F12 without antibiotic and incubated in a humidified incubator containing 5% CO_2 _at 37 °C for 2 h. Then miR-4270 inhibitor, a double-stranded RNA molecule that binds to mature hsa-miR-4270 (Sigma–Aldrich) and suppresses the mature hsa-miR-4270 (Sigma–Aldrich), was transfected into HepG2 cells by Lipofectamine 2000 (Invitrogen, Carlsbad, CA, USA) for 24 h or 48 h, according to the producer’s protocol. Following incubation, the treated and untreated cells were used for further experiments.

**Trypan blue staining assay**

HepG2 cells were plated into 24-well plates. The HepG2 cells at the confluency of 60- 70% were treated with different concentrations (0 as control, 10, 20, 30, 40, 50, 60, 70, 80, and 90 nM) of miR-4270 inhibitor for 24 h. The next day, cells were detached by trypsin, then stained using trypan blue 0.4% solution for three minutes and finally counted with a hemocytometer on a microscope (AX-71, Olympus Corporation, Shinjuku-Ku, Japan). The percentage of viable cells was calculated as follows: the total number of viable cells per ml of aliquot was divided into the total number of nonviable cells per ml of aliquot multiply 100. Assay was organized in three independent triplicate experiments (p < 0.05 vs. control group; ANOVA analysis).

**Gelatin zymography assay for MMP activity**

 HepG2 cells (2 × 10^5^ cells/well) were seeded on 12-well plates containing DMEM/F12 supplemented with 10% FBS in a humidified incubator (95% air and 5% CO_2_) at 37 °C. After 24 h, cells were treated with 30 nM of miR-4270 inhibitor and incubated at 37 °C for another 24 h. The following day, the culture media of the untreated and treated HepG2 cells were electrophoresed on 10% SDS-PAGE gels containing 1 mg/ml of gelatin (Merck, Germany). Next, the gel was incubated in a renaturation solution (2.5% Triton X-100, 5 mM CaCl_2_, and 1 mM ZnCl_2_ in 50 mM Tris-HCl, pH 7.4) at room temperature for 1 h, to renature MMP enzymes. Afterwards, the gel was incubated in activating buffer (5 mM CaCl_2_, 1 mM ZnCl_2_, and 50 mM Tris, pH 7.4) at 37 °C for 24 h. Finally, the gel was stained by Coomassie Blue dye (Merck, Germany) for 30 min and destained to observe MMP bonds.

**Wound healing assay**

Migration ability of untreated HepG2 cells as wells cells treated with miR-4270 inhibitorwas investigated by seeding the 4 × 10^5^ cells/well on 12-well plates containing the DMEM/F12 medium at 37°C. When the confluency of the cells reached ~80%, a scratch gap was made on the surface of the plates by a scraper. Then the treated HepG2 cells with 30 nM of miR-4270 inhibitor and untreated HepG2 cells in DMEM/F12 containing 5% FBS were incubated in 95% air and 5% CO_2 _at 37 °C for 48 h. Finally, migration of the cells into the scratch gap was evaluated under a microscope (AX-71, Olympus Corporation).

**Quantitative** **real-time RT-PCR**

RNA of the treated HepG2 cells with miR-4270 inhibitor and untreated HepG2 cells was extracted by RNeasy Plus Mini kit (Qiagen, USA) based on the manufacturer’s protocol. Complementary DNA synthesis was conducted using the QuantiTect1 Reverse Transcription Kit (Qiagen) in accordance with the protocol recommended by the manufacturer. The complementary DNA of miR-4270 was synthesized by stem-loop primer^[^^12^^]^. Primers of *MMP2* and *MMP9 *genes, as well as *GAPDH* as an endogenous control gene, were designed by online Primer-BLAST software (https://www.ncbi.nlm.nih.gov/tools/primer-blast/; Table 1). Quantitative gene expression was performed by the SYBR Premix Ex Taq II (Takara Bio Inc., Japan) in a Rotor-Gene 3000 System (Corbett Research, Australia). Data analysis of real-time RT-PCR was carried out by the 2^-ΔΔCt ^method.

**Table 1:** Primer sequences used in real-time RT-PCR

**Genes **	**Primers**	**Sequences (5'→3')**	**Primer length (bp)**	**Product length (bp)**	**Ta** **(°C)**
*GAPDH*	25	ACTCTGGTAAAGTGGATATTGTTGC	Sence	54	162
	21	GGAAGATGGTGATGGGATTTC	Antisence		
					
*MMP2*	18	CCTAGCACATGCAATACC	Sence	48	149
	19	CATGGTCTCGATGGTATTC	Antisence		
					
*MMP9*	18	CGCGCTGGGCTTAGATCA	Sence	55	129
	20	TCAGGGCGAGGACCATAGAG	Antisence		
					
*miR-4270*	50	GTCGTATCCAGTGCAGGGTCCGAGGTATTCGCACTGGATACGACGCCCTC	Reverse transcription	58	70
	18	TCAGGGAGTCAGGGGAGG	Sence		
	19	GAACATGTCTGCGTATCTC	Antisence		

**Gene target prediction of miR-4270**

 TargetScan (http://www.targetscan.org), and miRanda (https://cbio.mskcc.org/miRNA2003/ miranda.html) were used to predict the target genes of miR-4270 by considering *p *value threshold at *p *≤ 0.05. 

**Statistical analysis**

The results of all quantitative experiments were assessed by student’s t-test and one-way ANOVA. Results were displayed as the mean ± SEM. A *p *< 0.05 was considered as statistically significant.

## RESULTS

**I** **nfluence** **of hsa-miR-4270 inhibitor on** **the cell viability of HepG2 cells**

**Fig. 1:**
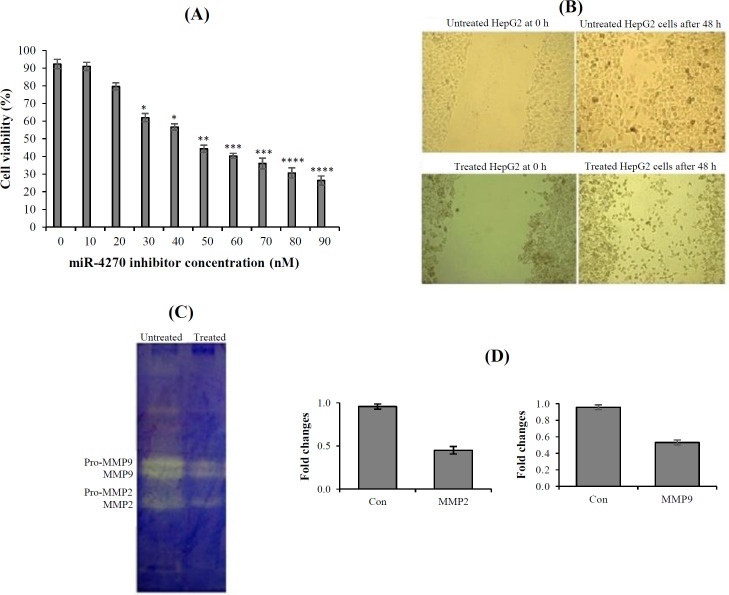
Trypan blue staining assay (A). MiR-4270 inhibitor decreased the cell viability of HepG2 cells in a concentration-dependent manner. (^*^p < 0.05, ^**^p < 0.005, ^***^p < 0.0005, and ^****^p < 0.00005 vs. control group, ANOVA analysis). (B) Zymography of HepG2 cells showing the decreased MMP2 and MMP9 activity in the treated compared to untreated cells. (C) Wound healing assay indicated that untreated cells had faster growth and movement into scratch gaps than the treated cells (magnification 100×). (D) The expression of *MMPs *genes evaluated by real-time RT-PCR (^*^p < 0.05 vs. control group, student’s t-test analysis).

Viability of the HepG2 cells treated with different concentrations of hsa-miR-4270 inhibitor (0, 10, 20, 30, 40, 50, 60, 70, 80, and 90 nM) was evaluated by trypan blue staining assay for 24 h. The results showed that hsa-miR-4270 inhibitor decreased the cell viability in a concentration-dependent manner (Fig. 1A). The IC_50_ value of the miR-4270 inhibitor in HepG2 cells was 47 ± 2.8 nM at 24 h. The concentration of 30 nM was chosen for hsa-miR-4270 inhibitor to treat HepG2 cells since this concentration had minimum cytotoxicity on the cells and the minimal significant concentration of has-miR-4270 inhibitor. At concentrations greater than 30 nM, the cell death increased and prevented the effect of hsa-miR-4270 inhibitor on cell migration and MMP activity.

**Migration** **and MMP activities in HepG2 cells**

The MMP2 and MMP9 activity was evaluated in the HepG2 cells treated with 30 nM of hsa-miR-4270 inhibitor using gelatin zymography. The results indicated a decline in the activity of both MMPs in the treated compared with the untreated HepG2 cells (Fig. 1B). To study the effect of hsa-miR-4270 inhibitor on the migration of the HepG2 cells, we scratched the surface of the plates sedded with HepG2 cells. Then the cells were treated with 30 nM of hsa-miR-4270 inhibitor. After 48 h, untreated cells showed faster growth and movement into scratch gaps than the cells treated with hsa-miR-4270 inhibitor (Fig. 1C). The results of wound healing assay indicated the inhibitory effect of hsa-miR-4270 inhibitor on the migration of the HepG2 cells.

**E** **ffect** **of** **hsa** **-miR-** **4270** **inhibitor on** ***MMP*** **gene expression in** **HepG2 cells**

 The effect of hsa-miR-4270 inhibitor on *MMP* genes expression in HepG2 cell line was assessed by real-time RT-PCR. The gene expression analysis of the cells treated with 30 nM of hsa-miR-4270 inhibitor for 24 h showed that the expression of *MMP2* and *MMP9 *decreased to 45% and 53%, as compared to the untreated cells, respectively (Fig. 1D). 

**Target genes of miR-4270**

 We used TargetScan and miRanda to find miR-4270 target genes. In this regard, the following genes were identified: *SMG6*,* VEGFB*,* TP53*, *KIAA1671*,* SMARCD1*,* CDH24*,* CDH1*, *WNT3A*,* TIAM1*,* CEMIP*,* MIEN1*,* ZBTB4*,* ELMO1*, *PRSS16*, and *ZDHHC9*. These genes are linked to various signaling pathways such as apoptosis, angiogenesis, migration, and mobility.

## DISCUSSION

 Investigation of the underlying mechanisms of different miRNAs in various human cancers have indicated that dysregulation of miRNA expression might influence cancer pathogenesis and therapy^[^^13^^,^^14^^]^. Several miRNAs have contrary roles in cancers based on the etiology of the disease. For instance, miR-186 acts as a tumor suppressor miRNA in endometrial cancer and squamous cell carcinomas. Interestingly, this miRNA acts as an oncomiR in some solid cancers, such as gastric, breast, and hepatocellular carcinoma^[^^15^^]^. Studies on the functions of miR-4270 in different cancers have introduced miR-4270 as a tumor suppressor miRNA in nasopharyngeal carcinoma and retinoblastoma, but as an oncomiR in Sertoli-cell-only syndrome, non-small-cell lung cancer, lung adenocarcinoma, and breast cancer^[^^16^^-^^22^^]^. 

 In this study, we evaluated the influence of miR-4270 suppression using hsa-miR-4270 inhibitor on MMP2 and MMP9 activity, as well as in vitro migration of HepG2 cells. Results of the cell viability and wound healing assays of the treated HepG2 cells with miR-4270 inhibitor represented the decreased proliferation and movement of these cells in comparison to the untreated cells. Findings of zymography and real-time RT-PCR experiments also indicated that the activity of MMP2 and MMP9 reduced at mRNA and protein levels in HepG2 cells treated with miR-4270 inhibitor as compared to the untreated HepG2 cells. Bioinformatics analysis of miR-4270 target genes revealed the role of miR-4270 in many signaling pathways, including angiogenesis, migration, and mobility. However, we found only a few experimental investigations on the role of miR-4270 in different cell signaling. Wang et al.^[^^23^^]^ have shown that miR-4270 directly targets SATB2, a key regulator of EMT signaling pathway in HCC. Wang and coworkers^[^^8^^]^ have displayed that the inhibition of miR-4270 reduces cell proliferation and apoptosis by inactivating NOTCH signaling pathway in Sertoli-cell-only syndrome patients. Moreover, Hao et al.^[^^17^^]^ have shown that miR-4270 directly targets *P53* gene in the human *neural progenitor* cells.

 The outcome of our study illustrated that miR-4270 could act as an oncogene in the HepG2 cells, although several previous studies have demonstrated a contradictory role for miR-4270 in HCC. Wang and coworkers^[^^23^^]^ have demonstrated that the expression of miR-4270 is downregulated in HCC patients, and mimic of miR-4270-5p suppresses the migration and cell proliferation of Huh7 and MHCC97, two HCC cell lines. Analysis of the microarray data has also indicated the downregulation of miR-4270 expression in HCC patients compared to the non-tumor tissues^[^^9^^]^. It is worth mentioning that HCC has various etiologies, such as non-alcoholic fatty liver disease, alcoholic liver disease, and different viral hepatitis, leading to tumorigenesis^[^^3^^,^^24^^]^. 

 Little data are known about the mechanism of action of miRNAs on HCC pathogenesis^[^^3^^,^^10^^,^^24^^,^^25^^]^. In this regard, we suggested that miRNAs played role through different mechanisms in various cell lines of HCC. In agreement with our findings, several other studies obtained inconsistent results on the mechanism of miR-4270 in different gastric cancer cell lines^[^^26^^-^^28^^]^. Shen et al.^[^^26^^]^ have reported that circ_0005556 is significantly upregulated in gastric cancer tissues, acts as a miR-4270 sponge and suppresses miR-4270 expression. They have also found that the downregulation of miR-4270 induces cell proliferation and metastasis in MGC-803 and MKN-28, two gastric cancer cell lines. However, Tokuhisa et al.^[27] ^have indicated that miR-4270 and four other miRNAs are upregulated in a gastric cancer cell line and gastric cancer patients. These investigations have uncovered the contradictory behaviors of miR-4270 in different cell lines of a cancer type and revealed that the function of miRNA in cancer might be related to pathogenesis and etiologies.

In summary, the findings of this study exhibited that miR-4270 suppression could reduce the activities of MMPs, in vitro migration of HepG2 cells as well as gene expression of *MMP2* and *MMP9 *in the cells. While there was inconsistency between our results and those of other investigations on the role of miR-4270 in HCC, outcomes of the present study could help find not only a mechanism of miR-4270 but also a new therapy approach for HCC. 

## DECLARATIONS

### Acknowledgments

 Authors thank to the Vice-Chancellor of Research Department of Shiraz University of Medical Sciences, Shiraz, Iran for their financial support.

### Ethical statement

 Not applicable.

### Data availability

 The raw data supporting the conclusions of this article are available from the authors upon reasonable request.

### Author contributions

 HA: designed and conducted the experiments and wrote and revised the manuscript; HG: accomplished gene expression and cell culture; MRF: contributed to the design of the study and reviewed the manuscript; MZ: accomplished zymography.

### Conflict of interest

 None declared.

### Funding/support

This work was supported by the Vice-Chancellor of the Research Department of Shiraz University of Medical Sciences, Shiraz, Iran under no. 18056.
